# Assessment of subjective refraction with a clinical adaptive optics visual simulator

**DOI:** 10.1016/j.jcrs.2018.08.022

**Published:** 2019-01

**Authors:** Lucía Hervella, Eloy A. Villegas, Pedro M. Prieto, Pablo Artal

**Affiliations:** 1 Laboratorio de Óptica, Universidad de Murcia, Murcia, Spain; 2 Departamento de Física, Universidad de Murcia, and Voptica SL, Murcia, Spain

## Abstract

**Purpose:**

To clinically validate an adaptive optics visual simulator (VAO) that measures subjective refraction and visual acuity.

**Setting:**

Optics Laboratory, University of Murcia, Murcia, Spain.

**Design:**

Prospective case series.

**Methods:**

Using the adaptive optics visual simulator, 2 examiners measured the subjective refraction and visual acuity in healthy eyes of volunteers; 1 examiner also used a trial frame as a gold standard. The interexaminer reproducibility and agreement with the gold standard were estimated using the following statistical parameters: limits of agreement from Bland-Altman analysis, significance between differences (*P* value), and intraclass correlation coefficient (ICC).

**Results:**

Seventy-six eyes of 38 volunteers were measured. Interexaminer reproducibility for subjective refraction was excellent (ICC ≥0.96; *P* > .05), with low 95% confidence interval (CI) values for the power vectors M (spherical equivalent of the given refractive error), J0 (Jackson cross-cylinder, axes at 180 degrees and 90 degrees), and J45 (Jackson cross-cylinder, axes at 45 degrees and 135 degrees) (±0.51 diopter [D], ±0.14 D, and ±0.14 D, respectively). No significant differences in subjective refraction and visual acuity were found between the visual simulator and gold standard (*P* > .05), with 95% CIs for M, J0, and J45 (subjective refraction) of ±0.67 D, ±0.14 D, and ±0.16 D, respectively, and a ±0.10 logarithm of the minimum angle of resolution (visual acuity).

**Conclusion:**

Subjective refraction results using the adaptive optics visual simulator agreed with those of the gold standard and can be used as the baseline for visual simulation of any optical corneal profile or intraocular lens design for refractive surgery patients.

Today, there are several surgical options for correcting refractive errors and presbyopia, such as corneal ablation and implantation of monofocal or multifocal intraocular lenses (IOLs). The optical design is usually selected based on results in clinical studies, commercial reports, and/or clinical experience. A simulation of visual performance with different optical profiles would allow practitioners to select the most appropriate solution for each patient. An adaptive optics visual simulator (VAO, Voptica SL) was recently introduced commercially for that purpose. The visual simulator performs objective measurements (wavefront aberrations and objective refraction), subjective refraction, and visual simulation of optical profiles.

At present, the determination of the refractive error of an eye is performed in 2 steps. The first is an objective refraction, which is followed by a subjective refraction based on the results of the first examination. The objective refraction can be measured by retinoscopy, autorefraction, or aberrometry. Retinoscopy is the oldest method and is still commonly used in clinical practice. However, this technique is slower than others and requires several years of training to become proficient in its use. Also, the accuracy of the cylinder axis measurement^1^ and the repeatability are poor.^2^ In contrast, refractometers are faster and do not require experienced operators. However, unlike retinoscopy^3^ and aberrometry,^4^, ^5^ autorefraction cannot assess the ocular media and thus cannot be used for certain purposes, such as early detection of cataracts or keratoconus.

In recent years, aberrometry has become a crucial tool in the clinical practice of ophthalmology and optometry. Aberrometers provide a clinical characterization of the ocular optics by measuring objective refraction,^6^, ^7^ from lower-order aberrations (LOAs), and higher-order aberrations (HOAs).^8^ Furthermore, aberrometers based on the Hartmann-Shack principle help detect ocular diseases, such as keratoconus and cataract, by analyzing HOAs and Hartmann-Shack images.^4^, ^5^

Although objective refraction can provide good visual outcomes, neural processing must also be considered. Therefore, subjective refinement should be performed to achieve the best visual acuity and optimum optical correction. At present, 2 older devices—trial frames and phoropters—are the only options for performing subjective refraction. They require a long room, at least 4 m, to allow measurement of far vision with relaxed accommodation. Also, the change in power, sphere, and cylinder is done mechanically by choosing a trial lens from a set incorporated in the phoropter or manually from a box with the trial-frame method.

Before a new measurement method or technique is used in clinical practice, it must be determined whether the measurements it gives are sufficiently close to those generated by the gold standard currently used. At present, the trial-frame and phoropter procedures are the gold standard; thus, their measurements are accepted as reference values^9^ against which new devices and procedures should be validated.

The purpose of this study was to validate the subjective refraction performed with the VAO adaptive optics visual simulator by determining the interexaminer reproducibility of subjective refraction between 2 operators and assessing the agreement of subjective refraction and visual acuity by comparing results with those using a trial frame as the gold standard.

## Participants and methods

This prospective study comprised eyes from healthy volunteers. It was approved by the Ethics Committee, University of Murcia. The research followed the tenets of the Declaration of Helsinki, and all participants provided written informed consent.

Inclusion criteria were no current ocular pathology, no history of ocular surgery, and astigmatism less than 3.0 diopters (D). Participants using ocular drugs that could affect vision were excluded. No mydriatic or cycloplegic agents were used in this study.

### Adaptive Optics Visual Simulator

The VAO adaptive optics visual simulator combines aberrometry and adaptive optics technology for the measurement of ocular aberrations and the correction or simulation of optical patterns.^10^, ^11^ The instrument is 89 cm long, 36 cm wide, and 56 cm high. According to the manufacturer, the device measures subjective refraction quicker than a phoropter or trial frame.^A^ In addition, this instrument can simulate any optical profile or design before refractive surgery using the subjective refraction as the baseline.

The setup of the simulator has been described.^10^, ^12^ It has a Hartmann-Shack wavefront sensor^13^ to measure HOAs and to obtain an objective refraction from LOA data. An organic light-emitting diode (LED) presents visual stimuli (optotypes) to the patient. A liquid crystal on silicon spatial light modulator^14^ allows placement of the stimuli at the required distance and modification of the optical pattern through which these stimuli are seen by the person being examined.^15^ Thus, the sphere and cylinder powers can be changed, not necessarily in sequential order, during the subjective refraction process.

### Examination Protocol and Measurements

This study used 2 examination protocols, 1 using the VAO adaptive optics visual simulator (visual simulator) and 1 using a trial frame (gold standard). Monocular refraction was performed using the visual simulator in both eyes and without cycloplegia. The visual-simulator protocol was performed by 2 experienced optometrists (L.H., E.A.V.); the results were used to assess interexaminer reproducibility of the subjective refraction and visual acuity measurements. One of the optometrists (L.H.) performed the gold-standard examination; the results were used to determine the agreement between the subjective refraction and visual acuity measurements and the visual-simulator measurements taken by the other optometrist (E.A.V.). Both examiners were masked to each other's results.

First, 3 consecutive objective refraction measurements from Hartmann-Shack images were taken using the visual simulator. In both protocols, the mean of those measurements was used as the starting point to refine the subjective refraction.

Instead of autorefractor or retinoscope, Hartmann-Shack images were used as a starting point for subjective refraction. This was done for 3 reasons. First, previous studies^16^, ^17^, ^18^, ^19^ found a high correlation between objective refractions estimated from Hartmann-Shack aberrometers, autorefractors, and retinoscopy. Second, the influence on final subjective refraction of small variations between the 3 methods was minimized by a high myopization and detailed refinement with Jackson's cross-cylinder in the gold-standard protocol. Third, subjective refinement of both examiners and in both protocols was independent. There was no interaction of the sphere refinement values. The cylinder values and orientation were adjusted with the Jackson cross-cylinder in the gold-standard protocol, while cylinder values from Hartmann-Shack refraction were not changed in the visual-simulator protocol.

In both protocols, the sphere was refined in steps of ±0.25 D after myopization was performed. In the gold-standard protocol, after the best sphere was identified, a Jackson cross-cylinder test was performed to refine the power and axis of the astigmatism in eyes with astigmatic objective refractions. In the visual-simulator protocol, the cylinder values from aberrometry refraction were not subjectively refined. Based on clinical standards, the endpoint of subjective refraction was the maximum plus that gave the best visual acuity. Three corrected distance visual acuities (CDVAs) with the subjective refraction were also measured. In both protocols, visual acuity was measured using tumbling E charts with 5 random letters per line and 0.10 logarithm of the minimum angle of resolution (logMAR) steps between lines up to −0.2 logMAR, following the same configuration of the Early Treatment Diabetic Retinopathy Study test. Letter-by-letter scoring was performed in which a score of 0.02 logMAR was given for every letter read correctly. The luminance of both displays was fixed in 80 candelas/m^2^ according to the International Organization of Standardization 8596:2009 standard.^20^ Ambient-light room illumination was kept constant throughout the measurements.

In the visual-simulator protocol, visual acuities were measured using the organic LED inside the instrument, which was placed at infinity. For the gold-standard protocol acuity measurements, a 13.3-inch QHD + screen (3200 pixel × 1800 pixel) of a Yoga 2 Pro laptop (Lenovo Group Ltd.) was used. The screen was placed at 4 m.

### Refraction Notation

To avoid the effect of cylinder axis orientation when comparing refraction data from different procedures and operators, the spherocylindrical refraction values were converted to vector notation (M, J0, and J45) using the following equations^21^:

$$\text{M}=\text{S}\;+\;\frac{\text{C}}{2}$$

$$\text{J}0\;=\;-\frac{\text{C}\;}{2}\times \;\cos\;2\text{α}$$

$$\text{J}45\;=\;-\;\frac{\text{C}}{2}\;\times \;\sin\;2\text{α}$$

where M is the spherical lens equal to the spherical equivalent (SE) of the given refractive error; S is the sphere; C is the negative cylindrical power; α is the axis; J0 is the Jackson cross-cylinder, axes at 180 degrees and 90 degrees; and J45 is the Jackson cross-cylinder, axes at 45 degrees and 135 degrees, representing oblique astigmatism.

### Statistical Analysis

Statistical analysis was performed using R Core Team software (2016, R Foundation for Statistical Computing). The Shapiro-Wilk test was used to assess normality of all variables analyzed. Nonparametric statistics were applied in non-normally distributed variables. Differences between variables were obtained using the Student *t* test for normally distributed variables and the Wilcoxon signed-rank test for non-normally distributed variables. All statistical tests were 2-tailed, and *P* values less than .05 were considered statistically significant.

Bland-Altman analysis^22^ was performed separately for the M, J0, and J45 power vectors and for visual acuities. From the standard deviation (SD) of differences between measurements, 75% and 95% confidence intervals (CIs) were calculated by multiplying the SD by 1.15 and 1.96, respectively. The 95% limits of agreement (LoA) between measurements were also calculated as the mean difference ±95% CI, providing a range in which 95% of the differences between measurements by the 2 methods were expected to fall.

Furthermore, the intraclass correlation coefficient (ICC)^23^ was estimated using the variance components from a 1-way analysis of variance. A correlation is considered excellent when the ICC values are between 0.75 and 1.00.^24^

There was a low correlation (ICC < 0.50) between the differences of all parameters in right eyes and left eyes, indicating that each eye could be considered independent. All statistical parameters in right eyes and left eyes were also estimated to confirm that the results would be not affected by the possible correlation of the 2 eyes of the same volunteer. There were no significant differences in parameters in all eyes between right eyes and left eyes. The differences in the 95% LoA were 0.05 D or less. Thus, all results given are right eyes and left eyes together.

The sample limits the degree of confidence.^25^ The sample size for the reproducibility and agreement measurements was the same: 76 eyes, so that the degree of confidence in the LoA was also the same: 16%.

## Results

The study included 76 eyes of 38 volunteers. The mean age of the 20 men and 18 women was 34.6 years ± 13.7 (SD) (range 21 to 72 years). The mean manifest spherical refractive error was −0.94 ± 2.26 D (range −5.00 to +4.50 D), and the mean cylinder was −0.57 ± 0.53 D (range −2.25 to 0.00 D).

### Interexaminer Reproducibility

**Figure 1:**
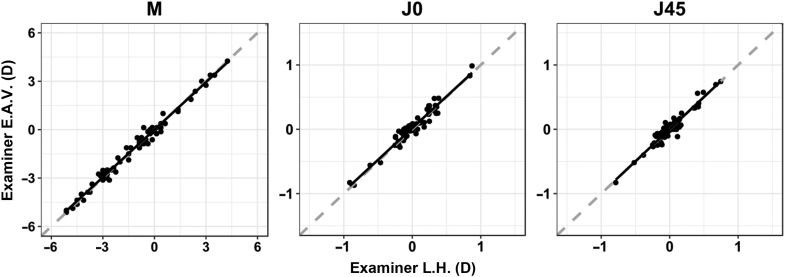
Linear correlation between vector refraction power values obtained by the 2 examiners with the visual simulator (M = spherical lens equal to the spherical equivalent of the given refractive error; J0 = Jackson cross-cylinder, axes at 180 degrees and 90 degrees; J45 = Jackson cross-cylinder, axes at 45 degrees and 135 degrees).

Concerning the correlation between the measurements by the 2 examiners taken with visual simulator, the best fitting line was very close to the line of equality for all refractive parameters, with high linear correlation coefficients (ICC >0.96) and no significant differences (*P* > .05) (Figure 1). Thus, the correlation between the results of the 2 examiners was excellent.

**Figure 2:**
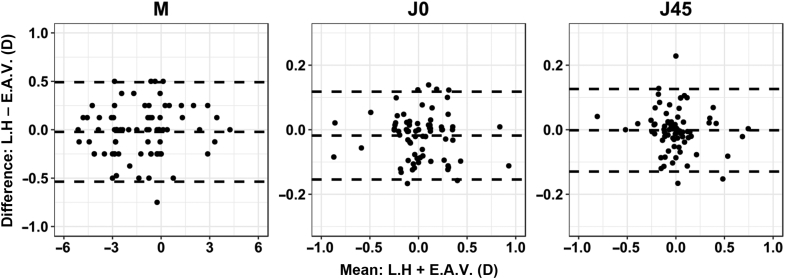
Differences in refractive power vectors between examiners (mean interexaminer difference [bias] and 95% LoA) (M = spherical lens equal to the spherical equivalent of the given refractive error; J0 = Jackson cross-cylinder, axes at 180 degrees and 90 degrees; J45 = Jackson cross-cylinder, axes at 45 degrees and 135 degrees; LoA = limits of agreement).

Figure 2 shows the Bland-Altman graphs of the refractive power vectors; that is, the differences between the 2 examiners as a function of the interexaminer mean value, suggesting good reproducibility between the 2 examiners. The mean difference (bias) for each refractive parameter was approximately zero, which indicates no consistent bias in the measurements. There was no tendency toward myopic or hyperopic shift (Figure 2). The 95% LoA ranged from 0.49 to −0.54 (CI, ±0.51 D), 0.12 to −0.15 (CI, ±0.14 D), and 0.13 to −0.13 D (CI, ±0.14 D) for M, J0, and J45, respectively. The 75% CI was 0.30 D or less for all parameters. Eighty-one percent of the differences in the SE (M) between examiners were within ±0.25 D. Moreover, more than 98% were within ±0.50 D, the limit often considered of clinical significance in clinical practice. All differences in cylinder powers were within ±0.25 D.

Furthermore, there was no significant difference in the CDVA measured by the 2 examiners with the visual simulator (*P* > .05). The mean difference was 0.01 ± 0.06 logMAR, and the 95% LoA ranged from −0.10 to 0.11 logMAR (CI, ±0.12). Approximately 78% of the differences were within ±0.05 logMAR and 95% within ± 0.10 logMAR; thus, most of the differences were approximately 1 line or less of 5 E letters.

### Agreement with the Gold Standard

**Figure 3:**
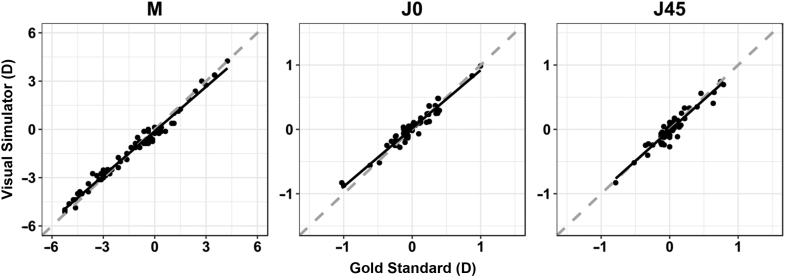
Linear correlation between vector refraction power values obtained with the visual simulator and gold standard (M = spherical lens equal to the spherical equivalent of the given refractive error; J0 = Jackson cross-cylinder, axes at 180 degrees and 90 degrees; J45 = Jackson cross-cylinder, axes at 45 degrees and 135 degrees).

There was a high linear correlation (ICC > 0.94) and no statistically significant differences (*P* > .05) between refractive power vectors measured with the visual simulator and the gold standard (Figure 3). In addition, the best linear fit was close to the line of equality for all refractive parameters, showing good equivalence between the 2 methods.

**Figure 4:**
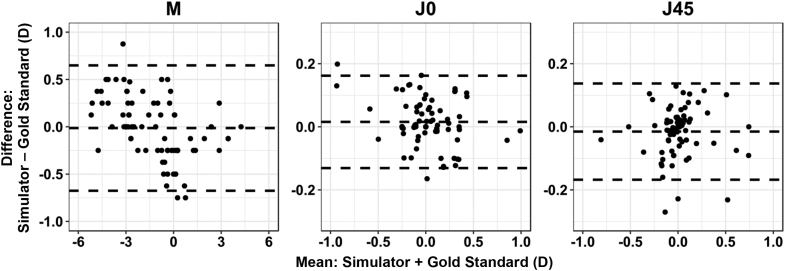
Differences in refractive power vectors between the visual simulator and the gold standard (mean difference [bias] and 95% LoA) (M = spherical lens equal to the spherical equivalent of the given refractive error; J0 = Jackson cross-cylinder, axes at 180 degrees and 90 degrees; J45 = Jackson cross-cylinder, axes at 45 degrees and 135 degrees; LoA = limits of agreement).

Figure 4 shows the Bland-Altman graphs of the differences in the mean refractive power vectors between visual simulator protocol and the gold standard protocol. The mean differences were close to zero for all 3 vectors. The 95% LoA ranged from 0.65 to −0.67 (CI, ±0.67 D), 0.16 to −0.13 (CI, ±0.14 D), and 0.14 to −0.17 D (CI, ±0.16 D) for M, J0, and J45, respectively. The 75% CI was below 0.39 D for all parameters.

A comparison of M values between the 2 protocols showed that 67.1% of the differences were within ±0.25 D and 93.4% were within ±0.50 D; in 6.6% of cases, the values differed by more than ±0.50 D. All differences in cylinder powers were within ±0.25 D.

There was a high linear correlation between the CDVA measurements taken by examiner E.A.V. with the visual simulator and measurements taken by examiner L.H. using the gold standard (ICC = 0.76), with no significant differences (*P* > .05) and a mean difference of 0.01 ± 0.05 logMAR. Individual differences were approximately 1 or below 1 line, with the 95% LoA ranging between −0.10 logMAR and +0.12 logMAR (CI, ±0.10). Overall, 70% of the differences were between ± 0.05 logMAR and 92% within ±0.10 logMAR.

## Discussion

This study evaluated measurements taken using the new VAO adaptive optics visual simulator and those taken using the gold standard (trial frame) in eyes of healthy individuals. Subjective refraction and visual acuity measurements taken with the visual simulator were validated, showing a good interexaminer reproducibility and agreement with the gold-standard method.

The low variability in subjective refraction values between examiners indicated the good reproducibility of the adaptive optics visual simulator, with results similar to or better than those in previous studies that used standard techniques. MacKenzie^26^ evaluated the subjective refraction measurements of the same patient performed by 40 optometrists and found a 95% CI of ±0.55 D for M and ±0.17 D for J0 and J45. Reinstein et al.^27^ found a 95% CI of ±0.44 D for M between surgeons and optometrists in 922 patients. Leinonen et al.^28^ compared 2 subjective refraction values obtained over a mean interval of 45 days by different examiners under the same clinical conditions. They found a 95% CI for the M vector of ±0.51 D between measurements in volunteers with a visual acuity of 0.15 logMAR or better. In a study of the precision of refraction from wavefront measurements compared with that of autorefraction and subjective refraction, Pesudovs et al.^29^ obtained higher 95% CI values for subjective refraction (±0.48 D, ±0.20 D, and ±0.13 D for M, J0, and J45, respectively), which is approximately double the values from autorefraction and lower-order wavefront refraction. In a study by Bullimore et al.,^30^ 2 optometrists measured the subjective refraction in eyes of 86 volunteers, finding a 95% LoA of −0.90 to +0.65 D, −0.37 to +0.39 D, and −0.31 to +0.31 D for M, J0, and J45, respectively.

Sheedy et al.^31^ measured the interexaminer reproducibility of subjective refraction between 2 sessions and 2 operators; the 95% LoA for M, J0, and J45 was −0.66 to +0.52 D, −0.40 to 0.34 D, and −0.25 to 0.21 D, respectively. In the present study, the 95% LoA were narrower (−0.54 to 0.49 D, −0.15 to 0.12 D, and −0.13 to 0.14 D, respectively) than in previous works.^31^ The mean interexaminer difference in the M values with the adaptive optics visual simulator were lower (−0.02 D) than that reported by Leinonen et al.^28^ for subjective conventional refraction (+0.05 D) and Bullimore et al.^30^ for automated subjective refraction (−0.12 D).

In our study, the agreement of cylinder power vectors (J0 and J45) between the adaptive optics visual simulator and the gold standard was similar to the reproducibility with the visual simulator (95% CI, ±0.14 D and ±0.16 D, respectively). However, the agreement for M was ±0.67 D, which is slightly worse. This is because the measurements in the reproducibility analysis were performed by both examiners using the visual simulator while the agreement was estimated using different techniques. However, our agreement outcomes were similar to or better than those of earlier comparisons of other methods used to measure refraction. Bennett el al.^32^ repeated refractive measurements with an aberrometer, an autorefractor, and manual subjective refraction, reporting 95% CI values between ±0.50 D and ±0.60 D.

Sheedy et al.^31^ compared the results of automated subjective refraction and subjective refraction performed by clinicians; the 95% CI for M, J0, and J45 was ± 0.76 D, ±0.33 D, and ±0.29 D, respectively. Hastings et al.^33^ reported 95% LoA of −1.82 to 0.86 D between objective refraction from a visual-image quality metric and subjective refraction with a phoropter. A study by Zadnik et al.^2^ comparing subjective refraction and autorefraction with paralyzed accommodation found 95% LoA for M of −1.26 to +0.94 D. This range is twice as wide as our outcomes without cycloplegia, indicating that in general autorefraction normally gives wider LoA than subjective refraction, even with paralyzed accommodation.

A review of the intraexaminer and interexaminer reliability of conventional subjective refraction by Goss and Grosvenor^34^ found that 95% of the differences were within ±0.50 D, similar to our outcomes of 98.0% for interexaminer reproducibility of the adaptive optics visual simulator and 93.4% for the agreement between the visual simulator and the gold standard.

Our visual acuity measurement outcomes were similar to those in earlier studies. Sheedy et al.^31^ reported a repeatability (expressed as the 95% CI) between visit 1 and visit 2 of approximately ±0.16 logMAR, similar to our findings of ± 0.12 logMAR for interexaminer reproducibility. The differences in visual acuity between the 2 methods used by Hastings et al.^33^ (visual Strehl ratio objective and subjective refraction) had a 95% CI of approximately ±0.08 logMAR, which is comparable to the 95% CI of ±0.10 logMAR between the visual simulator and gold standard in our study.

Results in some reports suggest that close-view instrument yields consistent bias toward over-minus or under-plus refraction compared with open-view instruments^35^ or subjective refraction. In our study, there were no significant differences in refraction measurements between the visual simulator and the gold standard, showing that accommodation had no affect.

Visual acuity and subjective refraction can be affected by several factors, including pupil size, ocular and general health, and changes in accommodation in the fixation state.^28^ The ability of different persons to discern dioptric differences varies from 0.12 D to 1.00 D.^36^

Finally, our findings suggest that the VAO adaptive optics visual simulator provides consistent and reproducible subjective refraction and that its measurements are comparable to those of the gold-standard technique in healthy eyes. Thus, subjective refraction found with the visual simulator can be used as the baseline to simulate any optical profile and to customize the selection of the ablation and the IOL design in refractive surgery.

**What Was Known**
• At present, subjective refraction is measured using trial lenses to refine objective refraction from retinoscopy or autorefractors.
• Optical profiles or designs selected for a refractive surgery are not based on the previous visual performance of the patient.

**What This Paper Adds**
• Subjective refraction with the adaptive optics visual simulator agreed with that using the gold-standard procedure.
• The adaptive optics visual simulator can be used to measure subjective refraction and serve as a baseline to simulate any refractive profile or design to customize ablation treatments or IOLs.
